# Time for sharing data to become routine: the seven excuses for not doing so are all invalid

**DOI:** 10.12688/f1000research.8422.1

**Published:** 2016-04-29

**Authors:** Richard Smith, Ian Roberts

**Affiliations:** 1 ICDDR, B, Dhaka, Bangladesh; 2 Former editor, BMJ, London, UK; 3 Faculty of Epidemiology and Population Health, London School of Hygiene & Tropical Medicine, London, UK; 4 Clinical Trials Unit, London School of Hygiene & Tropical Medicine, London, UK

**Keywords:** Data sharing, data analysis, data management, publishing

## Abstract

Data are more valuable than scientific papers but researchers are incentivised to publish papers not share data. Patients are the main beneficiaries of data sharing but researchers have several incentives not to share: others might use their data to get ahead in the academic rat race; they might be scooped; their results might not be replicable; competitors may reach different conclusions; their data management might be exposed as poor; patient confidentiality might be breached; and technical difficulties make sharing impossible. All of these barriers can be overcome and researchers should be rewarded for sharing data. Data sharing must become routine.

Good, well curated data are more valuable than the words authors write about them, but until now the main currency of science has been publications. With the World Wide Web sharing and publishing data is now possible, and researchers should be rewarded for doing so. Authors unfortunately have incentives not to share data and continue to find excuses for not doing so – but the excuses are poor. It’s time for data sharing to become routine.

## The value of data

Datasets are more valuable than papers because: they allow analyses to be replicated helping to avoid error, selective reporting and fraud; they can be used to answer other research questions; and they facilitate methodological research and the teaching and training of researchers. Papers, in contrast, rarely report the full data and are often “spun” to present results that flatter authors and please editors.

## Patients are the main beneficiaries of data sharing

The main beneficiaries of sharing data are patients, the people who as taxpayers fund most research. They clearly have an interest in both the right conclusion being reached and in maximum value being squeezed from every dataset. Unfortunately many others in the research system do not have the same interest in the “truth.”

If we consider a clinical trial or indeed any study with clinical implications then the prime interest of the patients is that the results are “true” and that clinicians use them to improve their well-being. This means that the analyses should be accurate and replicable. Sadly the producers of research have interests apart from truth: researchers want high impact papers; universities want the same and lots of publicity too; editors and publishers want “good” publications that increase their impact factor; and funders want to show “value for money,” which may means lots of publications regardless of their truth. Nobody is incentivised to share data, replicate results, and perhaps show the weak underbelly of science, which is why the scientific community has responded so poorly to allegations of misconduct ^1^.

By participating in clinical research patients make a gift to others, rather as those who give blood do. They and their gift, their data, should be treated with reverence. Their gift is not for individual researchers to use to advance their careers but for the wider scientific community and other patients. Their gift must be shared.

## The seven incentives not to share

Because they are measured primarily by how much and where they publish, researchers are strongly incentivised to publish, preferably in high impact journals. There are not the same incentives to share data. Indeed, there are seven incentives (or excuses) not to share.

Firstly, data are the base for research articles, and one anxiety for researchers is that others will use their data to produce publications without having to go to the trouble of gathering them. They will be disadvantaged in the academic rat race, although if everybody shared data they could benefit from using data from others.

Secondly, other researchers might scoop them, perhaps even prevent them from achieving publication in a high impact journal. Funders who require data sharing have responded to the anxiety of being scooped by allowing researchers to delay sharing their data. A better response would be to move away from “outsourcing” the judgement of the performance of researchers to publishers and for employers and funders to recognise that judging researchers is core business that should not be outsourced to the arbitrary and corrupted publishing process.

A third reason for not sharing data is a fear held by researchers that their conclusions will not be replicable. This is an ignoble reason because replicability is central to science. Some scientists may fear replication because they repeat experiments day after day and publish them only when they become “right.” This is unscientific and can lead to serious defects in the scientific evidence base.

One of us (IR) has made data from two large clinical trials available in the hope that somebody will replicate the analysis and confirm (or fail to confirm) the results ( https://ctu-app.lshtm.ac.uk/freebird/) ^2, 3^. Although the data have been used to answer many different questions, there has been no replication of the original trial results, probably because there is no incentive to do so - there ought to be. It surely makes economic sense for the millions spent on the trial to be backed up by the few thousands that would be needed to encourage replication. We hope that somebody will take up the challenge.

A fourth reason researchers may want to keep their data to themselves is to avoid their critics analysing the data and coming up with different or contrary results. Statisticians say that “if you torture the data they will confess,” but refusing to release data hands a victory to critics who will inevitably say “the researchers obviously have something to hide, they can’t support their conclusions.” Uncomfortable as it may be, it’s a better and more scientific strategy to enter “the market of ideas” and expect to show the correctness of your analysis and conclusions.

There is a legitimate worry about releasing data when researchers fear they may be sued. The problem here is that a battle in court is not a battle of evidence and data but a battle of showmen with a highly uncertain outcome. This is not a worry with most datasets, and perhaps when it is the data can be released in exchange for a legally binding commitment not to sue.

The authors of a major trial that showed the ineffectiveness of hydroxyethyl starch solutions for fluid resuscitation have declined to share their data ^4, 5^. They say that there have been “repeated efforts to discredit” by critics who want “to protect their commercial interests.” The authors have declined even to allow a reanalysis by a third party. This cannot be in the interest of patients, who clearly want to know whether the treatment is ineffective or not, but the authors may have a legitimate worry about legal action.

The fifth and perhaps worst reason for not releasing data is that data management is often poor and sharing the data may expose horrible weaknesses, flaws, and inconsistencies in the data. Sadly this may be the commonest but least declared reason for not sharing data. That some universities dedicate more resources to media relations than research governance is disturbing but not surprising. Making a big splash in the news can bolster grant income and student recruitment even when the informational content of the research is doubtful.

A sixth excuse for not sharing data that is available to those who do research with patients is patient confidentiality. One case of private information of a patient being exposed could, some researchers argue, bring data sharing to a halt. It is a “never event” that must be avoided even if huge benefits are foregone by not sharing data. Patient confidentiality must be guarded, and most of the time it’s easy to do so by anonymising data and removing data on, for example, place and time. It’s true that small risks remain because of rare conditions and events and because of “jigsawing” (combining datasets to break confidentiality), but these small risks can be explained to patients, who will almost always consent to their data being made available in anonymous form. With datasets that are already collected patients might be asked to give retrospective consent.

Patient confidentiality is the reason that authors of a controversial trial on treatment of chronic fatigue syndrome give for not sharing their data, but inevitably they look as if they are hiding something ^6, 7^.

The final and probably weakest excuse researchers give for not sharing data is “technical reasons.” But this is a lame excuse—other areas of science—for example, physics, astronomy, and engineering—have shared datasets far larger and more complex than those produced in biomedical research. There are no insurmountable technical reasons to sharing and publishing data.

## Reward authors for sharing data

Researchers should be rewarded not for publications but for producing large amounts of high quality data. Papers are a poor measure of the quantity or quality of research data. In terms of papers, a trial with 100 patients is the same as one with 10 000 patients, even though the informational content of the latter is 100 times the former. And despite the reverence for peer review, data quality is remarkably hard to judge from publications.

Funders of research and employers of researchers need to change the incentives for researchers to encourage data sharing, but researchers must also recognise the weakness of their excuses and contribute to the big advance in science that can come from sharing and publishing data.
