# Supplementary material for: Time for sharing data to become routine: the seven excuses for not doing so are all invalid
Source: F1000Res. 2016 Apr 29;5:781. [Version 1] doi: 10.12688/f1000research.8422.1 (PMC4909097; doi:10.12688/f1000research.8422.1)
Supplement: Peer review history

# Peer review: Referee response for version 1

**DOI:** 10.5256/f1000research.9066.r14294

**Published:** 2016-06-13

**Authors:** Gustav Nilsonne

**Affiliations:** 1 Department of Clinical Neuroscience, Karolinska Institute, Stockholm, Sweden

This opinion piece describes and refutes seven arguments against sharing research data. The authors focus on clinical trials, but their reasoning is applicable to research with human participants in general.

 In the ongoing conversation about open research data in scientific journals, arguments against open data are not always presented clearly and explicitly. The mere listing of counterarguments in a paper that can be referenced is therefore an important contribution.

 The authors refute each argument against data sharing in a clear and coherent manner and their counterarguments are a valuable resource for researchers debating open data.

 I have only one minor point of criticism: the statement in the last paragraph that a study with 10 000 participants has 100 times more information content than a study with 100 participants does not take into account the diminishing information content in consecutive dependent observations. I suggest this may be reworded.

I have read this submission. I believe that I have an appropriate level of expertise to confirm that it is of an acceptable scientific standard.

# Peer review: Referee response for version 1

**DOI:** 10.5256/f1000research.9066.r13824

**Published:** 2016-05-13

**Authors:** Heather M Goodare

**Affiliations:** 1 Edinburgh Health Forum, Edinburgh, UK

Thank you for asking me to comment on this paper.   I can only speak from the point of view of patients and carers. 

The main problem is that of confidentiality of data, and some patients are worried about this.  The authors acknowledge that this could be a problem (a sixth excuse).  Anonymising data is of course essential, but 'small risks remain'.  Remember the case of the anonymous male with back problems who was written about in an American medical journal and turned out (without too much detective work) to be President Kennedy?  Personally I don't care tuppence who knows that I have had breast cancer - it's in the public domain anyway.  But some conditions people would not wish to be known about: abortions, STD, some mental illnesses, and so on.  If data are anonymised that is usually sufficient safeguard, but in epidemiological studies unique postcodes are a giveaway.

I am a member of the Public Panel of the FARR Institute in Scotland, and we have debated the matter of Big Data at length.  We have a system here called SHARE, where if you are happy for your data to be used for research you sign a form, obtainable from your GP's surgery.  This also gives permission for the residue of blood samples taken for routine purposes to be used for research.  Most people are happy, but some are not, even given the guarantee of anonymity. 

However, this system gives permission for data culled from healthcare registries to be used for research: it does not as far as I know include data from trials already conducted.  This to me is a new idea, and it raises different issues.

FARR talks about 'safe havens' for data, so that personal details cannot be shared and anonymity is guaranteed.  It seems to me that if data already gathered for research are to be released to researchers other than the original investigators, this raises an entirely new issue.  It would mean that consent forms should be revised so that they take account of the possibility that data will be shared with others at a later date. 

It is important to make it clear that healthcare data are not the property of the researchers who have only borrowed them: they belong to the patient.  Therefore, if data are to be made more widely available, the patient needs to give consent.  This means that consent forms need to make this explicit, and all other data used for research, for instance epidemiological studies that do not require active co-operation from the patient, need to have blanket consent from patients, who should be encouraged to complete a SHARE form.

Personally, I like to know what researchers are going to do with my data.  My husband and I were ‘consulted’ as members of a patient reference group about a stroke trial (he has had a stroke), and we both felt that it should not have gone ahead: the rest of the patient group thought so too, but it went ahead anyway.  I don’t know how it got through Ethics.  The relevance for this paper is that patients do have a right to say what their data are going to be used for.  If they don’t approve of the trial, then they won’t let their data be used.  If they have given permission initially for a study that they approve of, and the proposal is to share the data further, should they not be given a say in what their data are to be used for subsequently?  Once Big Pharma get their hands on the data who knows what will become of it.

This makes the sharing of data more complicated, but I believe it should be done, and that this article needs to take account of these issues.

I also have some minor copy-edit suggestions:

 

- Abstract: “[…]competitors **might** reach different conclusions[…]”
- The seven incentives not to share: “[…]for employers and funders to recognise that judging researchers is core business that should not be outsourced to the arbitrary and **corrupt** publishing process.”
- The seven incentives not to share: “This cannot be in the interest of patients, who clearly want to know whether the treatment is **effective** or not […]”
- The seven incentives not to share: “There are no insurmountable technical reasons **for not **sharing and publishing data.”

I have read this submission. I believe that I have an appropriate level of expertise to confirm that it is of an acceptable scientific standard, however I have significant reservations, as outlined above.

# Peer review: Referee response for version 1

**DOI:** 10.5256/f1000research.9066.r13665

**Published:** 2016-05-05

**Authors:** Thomas Walley

**Affiliations:** 1 Department of Health Services Research, University of Liverpool, Liverpool, UK

Data sharing has been an expectation and indeed a contractual obligation for all research funded by NIHR, the research arm of the NHS, for many years. This has meant that bona fides researchers can request access to study data for defined proposes and with a suitable protocol, which should not be unreasonably withheld, e.g. for purposes of IPD meta-analysis. This is not open but controlled access to the data. The arbiter of what is reasonable access to the data falls to the researcher in the first instance, then to his/her host institute, but ultimately to the funder who held the contract.  

The recent consultation from the ICMJE ( http://www.nejm.org/doi/full/10.1056/NEJMe1515172 will probably translate into a requirement that data sets be made available in a more transparent way, usually by host institutions, in some form of as yet undefined registry.

Why not open access? Smith and Roberts consider some of these issues:

Ownership of the data: this (and responsibility for curation and archiving) rests with the institute but subject to the terms of the contract. Inevitably however, a researcher will feel a degree of proprietary protectiveness towards data sets. Most of us are not as altruistic in this regard as Smith and Roberts would like. Given the incentives that exist in academia, some respect for the intellectual property that the researcher has created is inevitable, and usually an agreement to access the data either in collaboration or with due acknowledgement is an acceptable outcome for all.

Risks of confidentiality: many studies are not of the 20000 patients size that Roberts has made available: smaller studies, with geographically defined recruitment may mean that the patient is potentially identifiable, especially if complex sets of data – often collected in smaller studies but less likely in larger - can also be accessed. Regrettably, there are people who seem to thrive on breaking open data like this: I think that patient confidentiality requires us to ensure that the data remains anonymous, best achieved by limited rather than open access.

Poor data handling: making data available to others is not without substantial cost, at a time when most researchers are planning to move on to another study: e.g. labelling the files from complex data sets in clear manner understandable to those who have not lived and breathed it for several years. Hence collaborative access is an easier and less expensive solution, where possible. Archiving the data also poses problems – who will take responsibility for converting data from old systems or software.

NIHR have established a contractual obligation, but like most other funders, has not yet provided the level of funding to make this possible (except on one occasion to Roberts), nor a vehicle similar to the GSK-led clinicalstudydatarequest.com to facilitate this.

None of this is to argue against the principles that Smith and Roberts put forward, but only to point out that achieving their worthy aims will not be easy or as quick as it might seem. NIHR like other funders continue to work to support this aim. As part of this, the NIHR journals library is also considering what constitutes publication: perhaps a somewhat selective journal article, a detailed monograph as has been our practice ( www.journalslibrary.nihr.ac.uk) or in the future, such a document with access to the data. These questions will not be quickly solved, and need much more debate to which this article by Smith and Roberts is a valuable contribution

I have read this submission. I believe that I have an appropriate level of expertise to confirm that it is of an acceptable scientific standard.

# Response

**Published:** 2016-05-10

**Authors:** Richard Smith

**Affiliations:** Patients Know Best Ltd, UK

I’m grateful to Tom for giving a rapid and useful on our paper. No doubt he is right that it will take a longer time than we would like for data sharing to become routine.

Incentives are fundamental. At the moment incentives reward keeping data, but we must change the incentives. We argue that the data are more valuable than the papers that arise from them, and so funders of research should be thinking hard about how to reward the production of high quality data. At the moment huge value is being lost from data being locked away, and data have a longer lifespan than papers. At the very least funders should be willing to meet the costs of data sharing that Tom identifies.

The confidentiality risk is, I fear, exaggerated. The obvious response is for researchers to get consent from participants for data to be shared at the same time as minimising the risk of exposure. It used to be that doctors did not get consent from patients for the sharing of case reports, but now they have to—and few patients refuse. The risk of exposure from participation in a trial is way below that of a case report.
